# GH Therapy in Chronic Heart Failure: A Systematic Review and Meta-analysis of Randomized Controlled Trials

**DOI:** 10.1210/clinem/dgae814

**Published:** 2024-11-21

**Authors:** Nikolaos Theodorakis, Georgios Feretzakis, Magdalini Kreouzi, Dimitrios Anagnostou, Christos Hitas, Vassilios S Verykios, Maria Nikolaou

**Affiliations:** Department of Cardiology & Heart Failure Outpatient Clinic, Sismanogleio-Amalia Fleming General Hospital, Melissia 15127, Greece; School of Medicine, National and Kapodistrian University of Athens, Athens 11527, Greece; School of Science and Technology, Hellenic Open University, Patras 26335, Greece; School of Science and Technology, Hellenic Open University, Patras 26335, Greece; Department of Internal Medicine & 65+ Clinic, Sismanogleio-Amalia Fleming General Hospital, Melissia 15127, Greece; Department of Cardiology & Heart Failure Outpatient Clinic, Sismanogleio-Amalia Fleming General Hospital, Melissia 15127, Greece; Department of Cardiology & Heart Failure Outpatient Clinic, Sismanogleio-Amalia Fleming General Hospital, Melissia 15127, Greece; School of Science and Technology, Hellenic Open University, Patras 26335, Greece; Department of Cardiology & Heart Failure Outpatient Clinic, Sismanogleio-Amalia Fleming General Hospital, Melissia 15127, Greece

**Keywords:** growth hormone therapy, insulin-like growth factor-1, heart failure, multiple hormonal deficiency syndrome, anabolic hormones, meta-analysis

## Abstract

**Context:**

Guideline-directed medical therapy of heart failure (HF) primarily targets neurohormonal activation. However, GH has emerged as a potential treatment for the multiple hormonal deficiency syndrome, which is associated with worse outcomes in HF.

**Objective:**

This study evaluates the efficacy and safety of GH therapy in HF.

**Data Sources:**

A systematic search was conducted in PubMed, Cochrane Library, and ClinicalTrials.gov, according to PRISMA guidelines

**Study Selection:**

Randomized, placebo-controlled trials studying GH therapy in adult HF patients were included. Of the 1184 initially identified records, 17 studies (1.4%) met the inclusion criteria.

**Data extraction:**

Two independent authors conducted the search, with any disagreements resolved by a third author. Study quality was assessed using predefined criteria, including randomization, blinding, and the presence of a placebo group.

**Data Synthesis:**

A random-effects model was applied due to heterogeneity across studies. GH therapy significantly improved left ventricular ejection fraction (+3.34%; 95% CI, 1.09-5.59; *P* = .0037), peak oxygen consumption (+2.84 mL/kg/min; 95% CI, 1.32-4.36; *P* = .0002), and New York Heart Association class (−0.44; 95% CI, −0.08 to −0.81; *P* = .023). GH therapy also reduced the composite of death, worsening HF or ventricular tachycardia by 41% (RR = .59; 95% CI, 0.39-0.90; *P* = .013). Subgroup analyses indicated that patients with ischemic cardiomyopathy, baseline ejection fraction ≥30%, and longer treatment duration experienced greater benefits.

**Conclusion:**

GH therapy demonstrated improvements in cardiac function, exercise capacity, and HF symptoms, along with a statistically significant trend toward improvements in hard endpoints. Event-driven trials are needed to validate these findings.

Heart failure (HF) is the leading cause of hospitalization and the third leading cause of cardiovascular mortality, accounting for 1 in 4 cardiovascular deaths in the general population (1). Neurohormonal activation, characterized by excessive stimulation of the sympathetic and renin-angiotensin-aldosterone systems, is a key driver of HF with reduced ejection fraction (HFrEF) and has shaped the development of most guideline-directed medical therapies (GDMT) for the condition (2). In addition to neurohormonal activation, a reduction in anabolic hormones, known as multiple hormonal deficiency syndrome (MHDS), has also been linked to HF progression and adverse events (3).

The TOSCA registry revealed that >90% of HF patients were deficient in at least 1 anabolic hormone (testosterone, dehydroepiandrosterone sulfate, IGF-I, or triiodothyronine), and more than two-thirds had deficiencies in 2 or more. The prevalence of IGF-I deficiency, based on age-adjusted cutoffs, was nearly 50%. More importantly, MHDS was associated with a significantly higher risk of hospitalization or death (3).

GH plays a crucial role in regulating various physiological processes. GH stimulates hepatic IGF-I secretion, which promotes protein synthesis, tissue repair, and cellular growth (4). Notably, there are various reports in the literature of local expression of GH receptors in the cardiac and vascular cells, signifying potential IGF-I-independent effects of GH in the cardiovascular system. Furthermore, local production of IGF-I by cardiac and vascular cells has been implicated in various physiological and pathophysiological processes (5). In the cardiovascular system, GH and IGF-I can increase myocardial mass and contractility, enhancing ventricular performance. They also reduce peripheral vascular resistance and improve endothelial function. Key noncardiac effects include stimulating lipolysis, increasing skeletal muscle mass and strength, enhancing bone density, and exerting insulin-like glycemic effects through IGF-I (4).

Recent meta-analyses of other anabolic hormones in chronic HF, such as thyroid hormone and testosterone therapy, have shown promising results (6-8). However, there is a lack of an up-to-date systematic review and meta-analysis of randomized controlled trials (RCTs) focused on GH therapy in HF. The available evidence is confined to 2 older meta-analyses published more than 15 years ago, which were not exclusively based RCTs and included open-label studies as well (9, 10).

The objective of this systematic review and meta-analysis is to provide updated evidence on the efficacy and safety of GH therapy in HF from placebo-controlled RCTs, thereby sparkling further research on this important topic.

## Materials and Methods

### Data Sources

A comprehensive search was conducted using the PubMed and Cochrane Library databases, as well as the ClinicalTrials.gov registry up to September 10, 2024, according to PRISMA guidelines. The search strategy included the combination “growth hormone” AND “heart failure” OR “HFrEF” OR “HFpEF” OR “HFmrEF.” The search was constrained to placebo-controlled RCTs published in English language including human subjects.

### Study Selection

All relevant publications were reviewed, and duplicate articles were removed. Additionally, the reference lists of selected articles were hand-searched for potentially relevant studies. All human studies were included if they met the following criteria: crossover or parallel placebo-controlled RCTs investigating the administration of GH in patients with HF, a follow-up period of at least 4 weeks, and participants aged 18 years or older. Studies were excluded if they met any of the following criteria: non-English language articles, animal studies, reviews, comments, replies, mechanism research, case reports, case series, observational studies, nonrandomized studies, or trials without a placebo group. Both the search and study selection were performed by 2 investigators; any disagreements were resolved by consensus with the involvement of a third author.

### Data Extraction

Two investigators independently extracted data. For each study, the following baseline information was collected and reported: first author, year of publication, sample size, trial duration, sex distribution, mean age (±SD), mean New York Heart Association (NYHA) class (±SD), mean left ventricular ejection fraction (LVEF) (±SD), mean baseline IGF-I levels (±SD), GH dose, and the following clinical outcomes (as reported):

- LVEF
- NHYA class
- Maximum oxygen consumption (VO2max)
- Peak work
- Exercise duration
- Interventricular septum diameter (IVSd)
- Left ventricular posterior Wall Diameter (LVPWd)
- Left ventricular end-systolic wall stress (LVESWS)
- Left ventricular end-diastolic volume (LVEDV)
- Left ventricular end-systolic volume (LVESV)
- Heart rate (HR)
- Systolic blood pressure (SBP)
- Diastolic blood pressure (DBP)
- Adverse events: death, worsening HF, ventricular tachycardia (VT), atrial fibrillation, and bradyarrhythmias

Study quality was rigorously assessed using predefined criteria that focused on key aspects of study design known to impact the internal validity of clinical research, including randomization, blinding, presence of placebo group, withdrawals and dropouts, and other sources of bias.

### Statistical Analysis

For this aggregate data meta-analysis, pooled baseline characteristics were first calculated for mean age (±SD), sex distribution, mean baseline LVEF (±SD), mean baseline NYHA class (±SD), pooled percentage of patients with ischemic cardiomyopathy (ICM), weekly GH dose and pooled change in IGF-I in the treatment arm. Individual effect sizes were calculated for both parallel and crossover trials. The mean difference (MD) was obtained by subtracting the mean change in the placebo group from the mean change in the treatment group in each trial. The SD of the change for both the treatment and control groups was calculated and used to compute the pooled SD for parallel trials, or the SD of the treatment-control difference for crossover trials. The standardized mean difference (SMD, or Cohen's d) was then determined by dividing the MD by the pooled SD for parallel trials, or by the SD of the treatment-control difference for crossover trials. The variance of each individual SMD was subsequently calculated.

To compute the global effect size (pooled SMD), individual effect sizes were weighted by the inverse of their variance using a fixed-effect model. The pooled SMD was then used to calculate the pooled MD and SD to provide interpretable results. The variance of the global effect size was used to generate the 95% CI and *P* values.

Heterogeneity was assessed using the Q and *I*² statistics. When significant heterogeneity was detected, a random-effects model was applied, incorporating the τ² estimate into the weighting of each individual SMD. In cases where the random-effects model was used, all results presented reflect the outcomes from the random-effects model exclusively. A forest plot was constructed to display the global effect sizes and their corresponding 95% CIs for each outcome.

Publication bias was assessed using both Egger's and Begg's tests. If publication bias was detected, a sensitivity analysis was planned by removing the studies contributing to the bias. However, this analysis would only be conducted if bias was identified.

To investigate potential sources of heterogeneity and assess the impact of study-level characteristics on effect sizes, performed subgroup analyses were performed. The data were split into groups according to the following characteristics: average weekly GH dose (<14 and ≥14 U/week), percentage of patients with ICM (≥50% or <50%), baseline LVEF (<30% or ≥30%), and GH treatment duration (≤3 months or >3 months). If 1 subgroup did not include any studies for a specific outcome, the subgroup analysis was not performed for that outcome. Forest plots were constructed to display the SMDs and corresponding 95% CIs for each outcome within both subgroups. Furthermore, interaction *P* values where calculated to assess for the presence of significant heterogeneity between subgroups.

Finally, the adverse events from all studies were pooled and risk ratios (RRs), their 95% CIs and *P* values were calculated for death, worsening HF, VT, atrial fibrillation, bradyarrhythmias, and the composite outcome of death or worsening HF or VT.

All *P*-values are expressed at 2 significant figures, unless *P* ≤ .0001. Statistical significance was set at *P* ≤ .05.

All data were first extracted into an Excel file, and statistical analyses were performed using Python version 3.12.3.

## Results

### Literature Search

A comprehensive literature search across PubMed, Cochrane Library, and ClinicalTrials.gov yielded 1184 records. After removing 137 duplicates, 1047 records were screened. Of these, 1013 records were excluded based on irrelevant titles or study types specified in the exclusion criteria (eg, literature reviews, nonrandomized studies). This left 34 relevant records to be assessed for eligibility through full-text review. An additional 17 records were excluded: 1 ongoing RCT, 1 terminated RCT without results, 1 suspended RCT, 1 RCT without a placebo group, 7 nonrandomized trials, 3 trials assessing the acute effects of GH therapy, 1 trial involving a mixed children/adult population with muscular dystrophies, and 2 trials not in English. As a result, 17 studies were included in the systematic review and meta-analysis according to the inclusion and exclusion criteria (11-27). Notably, the studies by Perrot et al (2001) and Osterziel et al (2000) are analyses of the population from the trial by Osterziel et al (1998). Although some reported outcomes were used, these studies were not treated as separate RCTs in the meta-analysis.

**Figure 1.**
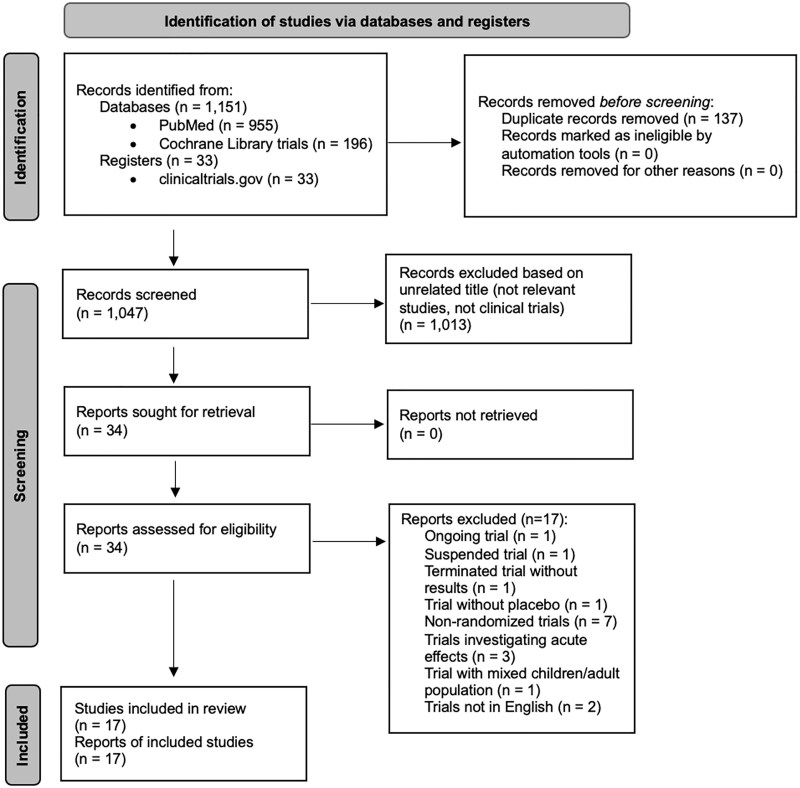
PRISMA 2020 flow diagram for systematic reviews.

The study selection and inclusion process followed the 2020 PRISMA guidelines for systematic reviews, as illustrated in the flowchart (Fig. 1).

### Baseline Characteristics

We synthesized data from 355 patients across the included studies. The studies by Cittadini et al (2009, 2013) exclusively enrolled patients with HF and baseline GH deficiency, whereas other studies included HF patients independent of GH/IGF-1 axis status. The demographic profiles and baseline characteristics are summarized in Table 1. The pooled baseline characteristics are as follows: mean age 58.9 ± 7.5 years, male sex 83%, mean baseline LVEF 30.2 ± 4.4%, mean baseline NYHA class 2.3 ± 0.5, and mean change in IGF-I in the treatment arms of 74% (from 15.6 ± 2.6 to 27.1 ± 3.7 nmol/L). Additionally, 48.4% of patients had ICM, whereas the remaining had dilated cardiomyopathy. GH was administered via subcutaneous injections at variable doses, with an average of 12.8 U/week.

**Table 1.** Baseline characteristics of the patients in the included studies

First author (year) (reference)	Sample size	Trial duration	Males	Mean age (±SD)	Mean NYHA (±SD)	Mean LVEF (±SD)	HF cause	Increase in mean IGF-1 (±SD) in the treatment arm	Weekly target dose
Amirpour et al (2021) (26)	n = 16 (placebo n = 8, GH n = 8)	12 months (3-month GH therapy)	100%	54 ± 2.7	NA	31.9 ± 3.6	ICM (100%)	NA	17.5 U
Karason et al (2020) (25)	n = 31 (placebo: n = 17, GH: n = 14)	9 months	95%	62.9 ± 2	2.7 ± 0.7	33.8 ± 8.5	ICM (100%)	100% (from 16.3 to 32.7 nmol/L)	29.4 U
Cittadini et al (2013) (24)	n = 31 (placebo: n = 14, GH: n = 17)	48 months	84%	62.9 ± 2	2.7 ± 0.1	32.5 ± 3.2	ICM (52%)	76.6% (from 12.3 ± 1.1 to 21.7 ± 1.4 nmol/L)	0.042 U/kg
Cittadini et al (2009) (23)	n = 56 (placebo: n = 28, GH: n = 28)	6 months	84%	62 ± 2.6	2.5 ± 0.7	32.5 ± 3.2	ICM (52%)	55.3% (from 12.3 ± 1.1 to 19.1 ± 1.4 nmol/L)	0.042 U/kg
Fazio et al (2007) (22)	n = 22 (placebo: n = 11, GH: n = 11)	3 months	69%	55.5 ± 10.5	2.4 ± 0.5	33 ± 4	nICM (57%)	103.4% (from 18.8 ± 4.6 to 38.3 ± 7.6 nmol/L)	14 U
Parissis et al (2005) (21)	n = 12 (crossover)	3 months	69%	53 ± 4	3 ± 0	24 ± 2	nICM (100%)	NA	14 U
van Thiel et al (2004) (20)	n = 19 (placebo: n = 10, GH: n = 9)	6 months	84%	65.4	2.6 ± 0.7	28 ± 7.3	ICM (100%)	23.6% (from 18.4 ± 6.1 to 21 ± 5.3 nmol/L)	14 U
Adamo-poulos et al (2003) (18)	n = 12 (crossover)	3 months	67%	50 ± 13.8	3 ± 0	23.6 ± 1.7	nICM (100%)	NA	14 U
Acevedo et al (2003) (19)	n = 19 (placebo: n = 9, GH: n = 10)	2 months	90%	57.7 ± 4.5	3 ± 0	<30	nICM (58%)	40.1%	0.245 U/kg
Adam-opoulos et al (2002) (17)	n = 10 (crossover)	3 months	70%	50 ± 4	3	24 ± 2	nICM (100%)	NA	14 U
Napoli et al (2002) (16)	n = 16 (placebo: n = 8, GH: n = 8)	3 months	75%	54.5 ± 11.3	2.3 ± 0.4	<40	nICM (69%)	106.3% (from 18.7 ± 3.7 to 38.6 ± 4.1 nmol/L)	14 U
Smit et al (2001) (15)	n = 19 (placebo: n = 10, GH: n = 9)	6 months	85%	65.5 ± 8.5	2.5 ± 0.7	27.9 ± 2.4	ICM (100%)	23.6% (from 18.4 ± 2 to 22.7 ± 1.8 nmol/L)	14 U
Perrot et al (2001), Osterziel et al (2000) and Osterziel et al (1998) (11, 13, 14)	n = 50 (placebo: n = 25, GH: n = 25)	3 months	86%	54 ± 10	2.3 ± 0.6	26	nICM (100%)	57.5% (from 17.5 to 27.6 nmol/L)	14 U
Spallarossa et al (1999) (12)	n = 20 (placebo: n = 10, GH: n = 10)	6 months	100%	62.1 ± 8	2.7 ± 0.4	32 ± 5.2	ICM (100%)	89% (from 13.1 ± 2.9 to 24.7 ± 6.8 nmol/L)	0.14 U/kg
Isgaard et al (1998) (10)	n = 22 (placebo: n = 11, GH: n = 11)	3 months	64%	60 ± 11.3	3 ± 0.5	29.7 ± 3.3	nICM (100%)	114.3% (from 22.9 to 49 nmol/L)	0.1 U/kg
Pooled characteristics	355	3 to 48 months	83%	58.9 ± 7.5	2.3 ± 0.5	30.2 ± 4.4	ICM (48.4%)	74% (from 15.6 ± 2.6 to 27.1 ± 3.7 nmol/L)	12.8 U

Abbreviations: HF, heart failure; ICM, ischemic cardiomyopathy; LVEF, left ventricular ejection fraction; nICM, nonischemic cardiomyopathy; NHYA, New York Heart Association.

### Efficacy Outcomes

Assessment using the Q statistic and *I*² statistic revealed significant heterogeneity for all outcomes across the different trials. Consequently, the analysis was switched from a fixed-effect model to a random-effects model for all outcomes, while exploring potential sources of this heterogeneity. Egger's and Begg's tests did not indicate significant publication bias across trials for any of the outcomes.

**Figure 2.**
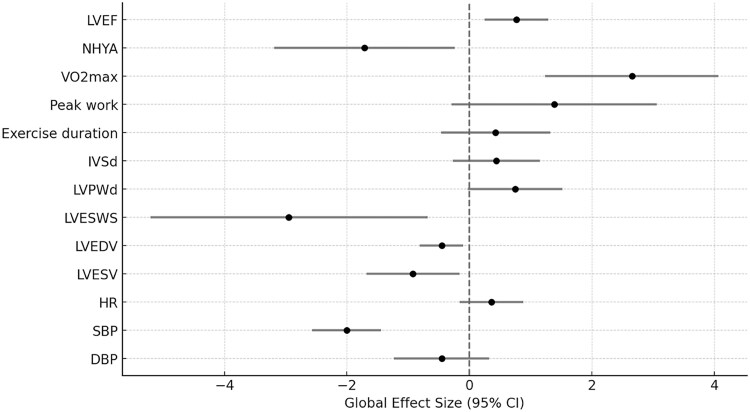
Forest plot of the global effect sizes and their 95% CIs for GH therapy in patients with heart failure. Abbreviations: DBP, diastolic blood pressure; HR, heart rate; IVSd, interventricular septum diameter; LVEF, left ventricular ejection fraction; LVEDV, left ventricular end-diastolic volume; LVESV, left ventricular end-systolic volume; LVESWS, left ventricular end-systolic wall stress; LVPWd, left ventricular posterior wall diameter; NHYA, New York Heart Association; SBP, systolic blood pressure; VO2max, maximum oxygen consumption.

GH treatment demonstrated a significant global effect size for the following outcomes: LVEF (0.77; 95% CI, 0.25-1.29; *P* = .0037), NYHA class (−1.71; 95% CI, −3.19 to −0.24; *P* = .023), VO2max (2.66; 95% CI, 1.24-4.07; *P* = .0002), LVESWS (−2.95; 95% CI, −5.21 to −0.68; *P* = .011), LVEDV (−0.45; 95% CI, −0.81 to −0.10; *P* = .012), LVESV (−0.92; 95% CI, −1.68 to −0.16; *P* = .018), and SBP (−2.0; 95% CI, −2.57 to −1.44; *P* ≤ .0001). However, global effect sizes did not reach statistical significance for peak work (1.39; 95% CI, −0.29 to 3.06; *P* = .1), exercise duration (0.43; 95% CI, −0.46 to 1.32; *P* = .35), IVSd (0.44; 95% CI, −0.27 to 1.15; *P* = .22), LVPWd (0.75; 95% CI, −0.03 to 1.52; *P* = .06), HR (0.36; 95% CI, −0.16 to 0.88; *P* = .18), and DBP (−0.45; 95% CI, −1.23 to 0.32; *P* = .25). A summary of the global effect sizes and their 95% CIs is presented in Table 2 and illustrated in a forest plot in Fig. 2.

**Table 2.** Summary of the global effect sizes and their 95% CIs

Parameters	Trials (no.)	Patients (no.)	Pooled MD (SD)	Pooled SMD	95% CI (lower)	95% CI (upper)
LVEF	13	329	3.34% (6.60)	0.77	0.25	1.29
NHYA class	6	182	−0.44 (0.3)	−1.71	−3.19	−0.24
VO2max	7	162	2.84 mL/kg/min (2.33)	2.66	1.24	4.07
Peak work	4	131	16.7 W (37.5)	1.39	−0.29	3.06
Exercise duration	6	189	43.4 seconds (145.1)	0.43	−0.46	1.32
IVSd	5	164	0.37 mm (1.11)	0.44	−0.27	1.15
LVPWd	7	188	0.46 mm (1.73)	0.75	−0.03	1.52
LVESWS	6	155	−116.6 kdyn/cm² (35.12)	−2.95	−5.21	−0.68
LVEDV	11	293	−20 mL (64.9)	−0.45	−0.81	−0.10
LVESV	10	271	−22 mL (52.7)	−0.92	−1.68	−0.16
HR	7	204	1.26 beats/min (7.3)	0.36	−0.16	0.88
SBP	6	154	−7.3 mm Hg (4.6)	−2.00	−2.57	−1.44
DBP	6	154	−2.7 mm Hg (5)	−0.45	−1.23	0.32

Abbreviations: DBP, diastolic blood pressure; HR, heart rate; ICM, ischemic cardiomyopathy; IVSd, interventricular septum diameter; LVEF, left ventricular ejection fraction; LVEDV, left ventricular end-diastolic volume; LVESV, left ventricular end-systolic volume; LVESWS, left ventricular end-systolic wall stress; LVPWd, left ventricular posterior wall diameter; MD, mean difference; nICM, nonischemic cardiomyopathy; NHYA, New York Heart Association; SBP, systolic blood pressure; SMD, standardized mean difference; VO2max, maximum oxygen consumption.

### Adverse Events

**Figure 3.**
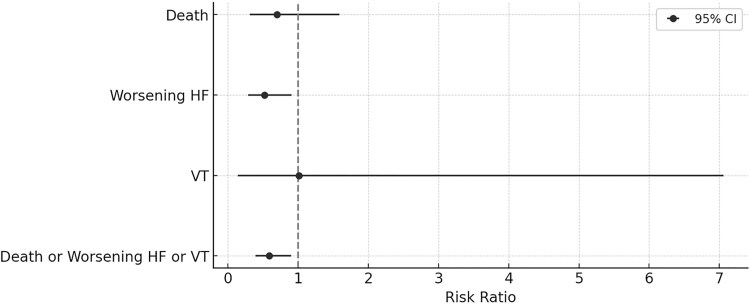
Forest plot of the pooled risk ratios and their 95% CIs regarding adverse outcomes during GH therapy in patients with heart failure. Abbreviations: HF, heart failure; VT, ventricular tachycardia.

Regarding pooled adverse events, deaths occurred in 5.1% of patients (GH group) and 7.3% of patients (placebo group), worsening HF in 9% of patients (GH group) and 17.4% of patients (placebo group). Furthermore, VT occurred equally, in 1.1% of patients in GH and placebo groups. The calculation of RRs for the pooled adverse events showed that GH therapy was associated with a statistically significant reduction in the risk of worsening HF (RR = 0.52; 95% CI, 0.29-0.91; *P* = .025) and the composite outcome of death, worsening HF failure, or VT (RR = 0.59; 95% CI, 0.39-0.90; *P* = .013) compared to placebo. The adverse events, their RRs, and their 95% CIs are presented in Table 3 and the forest plots are illustrated in Fig. 3.

**Table 3.** Summary of the pooled adverse events

Adverse outcomes	GH group	Placebo group	Risk ratios
Death	9	13	0.70 (95% CI, 0.31-1.59)
Worsening HF	16	31	0.52 (95% CI, 0.29-0.91)
VT	2	2	1.01 (95% CI, 0.14-7.06)
Death or worsening HF or VT	27	46	0.59 (95% CI, 0.39-0.90)
AF	0	4	Undefined
Bradyarrhythmias	0	1	Undefined

Abbreviations: HF, heart failure; VT, ventricular tachycardia.

### Subgroup Analyses

**Figure 4.**
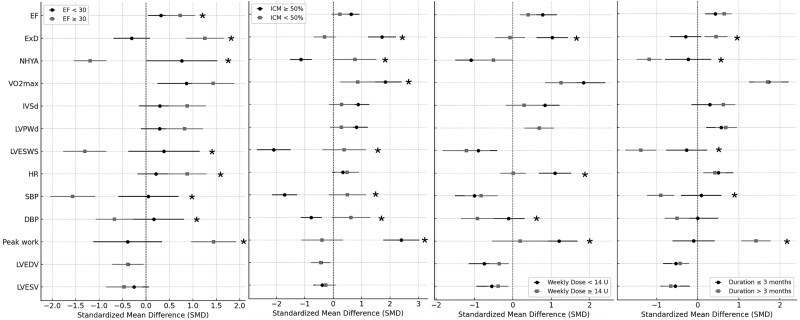
Forest plots of subgroup analyses illustrating the global effect sizes and their 95% CI according to baseline LVEF, cause of HF, dose of GH, and duration of GH therapy. Comparisons with an interaction *P* value ≤ .05 are marked with an asterisk. Abbreviations: DBP, diastolic blood pressure; HR, heart rate; IVSd, interventricular septum diameter; LVEF, left ventricular ejection fraction; LVEDV, left ventricular end-diastolic volume; LVESV, left ventricular end-systolic volume; LVESWS, left ventricular end-systolic wall stress; LVPWd, left ventricular posterior wall diameter; NHYA, New York Heart Association; SBP, systolic blood pressure; SMD, standardized mean difference; VO2max, maximum oxygen consumption.

To refine the understanding of GH's effects and investigate for the causes of between-study heterogeneity, subgroup analyses were performed to avoid bias. Because of the small number of studies in each subgroup, the study by Cittadini et al (2013) was excluded from the first 3 subgroup analyses because its follow-up duration was 48 months. Additionally, the study by Napoli et al (2002) was excluded from the baseline LVEF subgroup analyses from the lack of reported average LVEF. Subgroup analyses revealed significant differences in various outcomes. Specifically, studies with a mean baseline LVEF ≥30% demonstrated significantly larger improvements in LVEF, exercise duration, and peak work, along with greater reductions in NYHA class, LVESWS, and SBP/DBP. Additionally, studies in which ≥50% of patients had ICM showed significantly larger increases in exercise duration, VO2max, and peak work, as well as larger decreases in NYHA class, LVESWS, and SBP/DBP. Studies with a mean GH treatment duration of more than 3 months also showed significantly larger improvements in exercise duration and peak work, along with greater reductions in NYHA class, LVESWS, and SBP. The results of the subgroup analyses are illustrated in the forest plots in Fig. 4.

## Discussion

The physiological effects of GH, supported by evidence from preclinical studies, suggest the potential benefit of GH therapy in patients with HF (4). Although many RCTs have demonstrated positive results in improving symptoms, exercise capacity, and cardiac function, their small sample sizes have limited the ability to draw definitive conclusions. This meta-analysis, by pooling these outcomes, provides stronger evidence regarding the potential efficacy and safety of GH therapy in HF.

This meta-analysis demonstrated statistically significant improvements in left ventricular function, including increases in LVEF, decreases in LVEDV, and decreases in LVESWS, along with significant improvements in symptoms and exercise capacity, such as decreases in NYHA class and increases in VO2max. Despite the positive outcomes for VO2max, statistical significance was not achieved for peak work and exercise duration, although a nonsignificant trend toward improvement was observed.

Another key observation was that GH therapy did not affect resting HR, which is critical for HF patients, as both tachycardia and bradycardia can compromise the hemodynamic profile. Additionally, bradycardia may limit the ability to achieve the target dose of β blockers, a cornerstone of GDMT for HFrEF. Furthermore, GH therapy did not affect DBP, which is important because coronary flow and myocardial oxygen supply primarily occur during diastole. However, GH therapy did result in a statistically significant decrease in SBP by approximately 7 mm Hg. This reduction should be confirmed in larger future trials because it could pose a challenge in clinical application, given that many patients with HFrEF are already hypotensive, particularly in the context of GDMT.

The presence of significant between-study heterogeneity necessitates cautious interpretation of these findings, despite the use of a random-effects model. Subgroup analyses revealed significant trends among specific groups, which may be the primary drivers of this heterogeneity and offer further insights into the effects of GH in HF. These findings suggest that the greatest benefits in cardiac function, symptoms, and exercise capacity may be achieved with prolonged GH administration, particularly in patients with ICM and a baseline LVEF ≥30%. Notably, in the study by Cittadini et al (2013), the treatment effect on LVEF was substantial after 4 years, with a significant 10% increase in the GH group compared to placebo. This indicates that the effects of GH therapy in HF might have a time-dependent or duration-dependent component, where longer treatment durations yield more pronounced benefits. Another important observation from the studies by Karason (2020) and Amirpour et al (2021) was that the positive effects of GH administration diminished after cessation of treatment. These findings suggest that the beneficial effects of GH therapy may persist only during active administration.

Another interesting and unexpected finding was that studies with a mean GH dose of less than 14 U/week showed more significant increases in exercise duration and peak work, along with smaller decreases in DBP. This highlights the need for future studies to assess the optimal dose of GH therapy in HF, considering patient characteristics and baseline levels. It also suggests that moderate GH doses may be more effective than very high doses.

Regarding adverse events, it is important to note that the risk of VT did not differ between GH and control groups, which is particularly important because there are concerns regarding the potential of GH to cause malignant arrhythmias. Furthermore, although of included studies were not event-driven, the pooled analysis revealed significantly lower risk of worsening HF (by 48%), as well as a reduced risk for a composite outcome (death, worsening HF, or VT) (by 41%) in patients receiving GH compared to placebo. Although these findings are promising, they should be interpreted with caution, and future event-driven RCTs are needed to assess if GH therapy can improve hard endpoints in HF patients.

Mechanistic studies further support the findings of this meta-analysis, as GH has shown significant proliferative and antiapoptotic effects in the hearts of mice [4]. Additionally, recent research has demonstrated that GHRH not only stimulates GH secretion but also exerts direct protective effects on skeletal muscle and cardiac myocytes by promoting cell survival and inhibiting apoptosis through receptor-mediated mechanisms (28). Furthermore, preclinical studies have revealed that activation of the GHRH receptor signaling pathway can effectively prevent and reverse diastolic dysfunction, fibrosis, and cardiomyocyte hypertrophy, which are key features of HF with preserved ejection fraction (29). These findings suggest that GHRH administration represents a promising therapeutic avenue that warrants further investigation in HF management, regardless of LVEF.

A recent cohort study of older adults from the Cardiovascular Health Study found that components of the GH/IGF axis, particularly IGF binding protein-1 (IGFBP-1) and ghrelin, are predictive of adverse health outcomes and mortality. Elevated levels of these proteins, especially in the postprandial state, were associated with increased risks of cardiovascular events, hip fractures, and all-cause mortality. The inverse relationship between IGF-I and IGFBP-1 is especially significant: as IGFBP-1 levels increase, the bioavailability of free IGF-I declines, reducing its anabolic and metabolic effects (30). These findings suggest that elevated IGFBP-1 levels could serve as a biomarker to guide GH or GHRH therapy or other interventions aimed at lowering IGFBP-1. Future studies should investigate the potential role of measuring IGFBP-1 in HF, assessing its prognostic value and its association with the effects of GH treatment.

This meta-analysis has notable strengths. First, it includes only double-blinded placebo-controlled RCTs, which provide the highest level of evidence for evaluating the efficacy of interventions. The exclusion of nonrandomized studies, open-label trials, and observational studies ensures that the results are robust and minimizes bias. Second, in the presence of severe heterogeneity, there was a switch from fixed-effect model to random-effect mode, whereas subgroup analyses were performed to assess for causes of this heterogeneity. Third, the absence of publication bias supports the validity of the results. Finally, the systematic review follows PRISMA guidelines, and the methodology of meta-analysis are described in a way that allows replication of the study.

The limitations of this meta-analysis should be acknowledged. First, although random-effects models were applied to account for heterogeneity, the significant variability in study designs, populations, and intervention protocols across the included trials requires cautious interpretation of the pooled results. Second, the relatively small number of trials for certain outcomes may have reduced the statistical power to detect smaller effects, potentially increasing the risk of type II errors. Third, some studies did not report all outcomes of interest, limiting the robustness of findings for those specific outcomes. Additionally, although subgroup analyses provide valuable insights, the small number of studies in certain subgroups increases the risk of both type I errors and type II errors. A final important limitation concerns the analysis of pooled adverse events. These adverse outcomes were not the primary endpoints of the included studies, which may have led to inconsistencies in how events were reported across trials, introducing potential reporting bias. Additionally, most individual trials were not powered to detect differences in these adverse events, resulting in wide CIs and the possibility of random variation, especially for rare outcomes such as VT. Moreover, significant heterogeneity in study design, patient populations, and outcome definitions may limit the generalizability of the pooled adverse event estimates. As a result, future event-driven RCTs are necessary for a robust investigation of hard clinical endpoints.

In conclusion, this meta-analysis demonstrated that GH therapy may improve cardiovascular function, HF symptoms, and functional capacity in patients with HFrEF. Additionally, there was a trend toward a reduction in adverse events, including worsening HF and the composite outcome of death, worsening HF, or VT. However, these findings should be interpreted with caution because of the limitations in the available evidence. Future event-driven RCTs are necessary to fully assess the role of GH therapy as a potential GDMT for managing MHDS in HF.

## Data Availability

Some or all datasets generated during and/or analyzed during the current study are not publicly available but are available from the corresponding author on reasonable request.

## Abbreviations

DBP: diastolic blood pressure
GDMT: guideline-directed medical therapies
HF: heart failure
HFrEF: heart failure with reduced ejection fraction
HR: heart rate
ICM: ischemic cardiomyopathy
IVSd: interventricular septum diameter
LVEF: left ventricular ejection fraction
LVEDV: left ventricular end-diastolic volume
LVESV: left ventricular end-systolic volume
LVESWS: left ventricular end-systolic wall stress
LVPWd: left ventricular posterior wall diameter
MD: mean difference
MHDS: multiple hormonal deficiency syndrome
NHYA: New York Heart Association
RCT: randomized controlled trial
RR: risk ratio
SBP: systolic blood pressure
SMD: standardized mean difference
VO2max: maximum oxygen consumption
VT: ventricular tachycardia
